# Hybrid Printing Method of Polymer and Continuous Fiber-Reinforced Thermoplastic Composites (CFRTPCs) for Pipes through Double-Nozzle Five-Axis Printer

**DOI:** 10.3390/polym14040819

**Published:** 2022-02-20

**Authors:** Haiguang Zhang, Xu Lei, Qingxi Hu, Shichao Wu, Mohamed Aburaia, Joamin Gonzalez-Gutierrez, Herfried Lammer

**Affiliations:** 1 Rapid Manufacturing Engineering Center, Mechatronic Engineering and Automation of Shanghai University, Shanghai 200444, China; YamaS@shu.edu.cn (X.L.); huqingxi@shu.edu.cn (Q.H.); seedo@i.shu.edu.cn (S.W.); 2 Shanghai Key Laboratory of Intelligent Manufacturing and Robotics, Shanghai University, Shanghai 200072, China; 3 National Demonstration Center for Experimental Engineering Training Education, Shanghai University, Shanghai 200444, China; 4 Competence Center Digital Manufacturing and Robotics, University of Applied Science Technikum Wien, Höchstädtplatz 6, 1200 Wien, Austria; aburaia@technikum-wien.at; 5 Institute of Polymer Processing, Montanuniversitaet Leoben, Otto Gloeckel-Strasse 2, 8700 Leoben, Austria; joamin.gonzalez-gutierrez@unileoben.ac.at; 6 Kompetenzzentrum Holz GmbH, Altenberger Straße 69, 4040 Linz, Austria; h.lammer@wood-kplus.at

**Keywords:** 3D printing, continuous fiber-reinforced, 5-axis printer, printing path

## Abstract

The most widely used 3D process, fused deposition modeling (FDM), has insufficient interlayer adhesion due to its layer-by-layer forming method. A support material is also essential for the hollow parts and cantilevers. Moreover, the polymer materials used have limited mechanical properties. These issues have restricted the application of FDM in high-performance fields. Continuous fiber-reinforced thermoplastic composites (CFRTPCs) have high mechanical properties and have recently become the focus of research in the field of 3D printing. This paper, using pipe parts as an example, proposes a hybrid of pure polymer (pure PLA used) and CFRTPC (flax fiber pre-impregnated filament) material to develop a printing method based on the outstanding mechanical properties of CFRTPC material. After studying the printing path planning algorithm, the CFRTPC filament is laid along the axial and radial directions on the surface of the polymer base to improve the printed parts’ properties. The method feasibility and algorithm accuracy are verified through the development of five-axis printing equipment with a double nozzle. Through the printed sample’s tensile, compression and bending tests, the results show that the tensile, compressive and bending properties of PLA pipe can be significantly enhanced by laying CFRTPC filament along the axial and radial directions of the pipe. To summarize, the introduction of CFRTPCs greatly improved the mechanical properties of the printed parts, and the implementation of our method provides an effective way to solve the defects of the FDM process.

## 1. Introduction

Three-dimensional printing (3DP), also called additive manufacturing (AM), plays a key role in high-tech areas such as bioengineering [1] and aerospace [2] and is increasingly gaining attention. One of the most popular and widely used AM processes is fused deposition modeling (FDM) [3]. Polylactic acid (PLA) and Acrylonitrile Butadiene Styrene (ABS) are the most commonly used filaments in FDM because they are cheap, readily available and environmentally friendly. However, with increasing demands on product performance, high-performance plastics can also meet the increased requirements, but fiber materials perform even better. People are therefore turning their attention to composites in order to improve filament performance by adding fillers. CFRTPCs are currently the focus of research due to their excellent mechanical and chemical performances and potential light-weight structures [4]. In fiber performance tests, Tekinalp et al. [5] composited carbon fiber with ABS. They found that the tensile strength and tensile modulus of the fabricated test specimen increased by 115% and 700%, respectively, compared to traditional injection-molded composites. Tian et al. [6,7] studied a combination of continuous carbon fiber and PLA, and the flexural strength and modules reached 335 MPa and 30 GPa. Yao et al. [8] investigated the effect of polyethylene terephthalate (PET) fiber on PLA and found that the sample’s tensile strength reached 139 MPa after PET reinforcement. In addition, Yao et al. [9], Zhang et al. [10], Hao et al. [11], and Hu et al. [12] also carried out similar studies, which prove that various continuous fibers strengthen the mechanical properties of polymer materials to different degrees.

CFRTPC printing generally includes a single-step or double-step method. Luan et al. [13] used one extruder and two filament inputs, and then impregnated the fiber with PLA at the heating chamber, which allows the composite and printing processes to occur simultaneously; thus, it is called a single-step method. Although the mechanical properties of composites printed by this method are enhanced, it is difficult to fully impregnate the fiber with polymer, and both inter-layer and intra-layer voids can appear. Kentaro Sugiyama et al. [14] used one extruder and one prepared fiber for conventional FDM printing, meaning that continuous fibers pre-impregnated with polymers were prepared in advance before being printed through a print head. This double-step process is relatively complicated, but it can significantly improve the impregnation effect of the fibers and reduce inter-layer pores. In view of the above, this paper uses the double-step method to print CFRTPCs.

However, regardless of whether the single-step or double-step method is used to print CFRTPCs’ filament-forming printed parts, the tensile strength along the *Z*-axis of the printed parts is weaker than that of a pure PLA printing object. Analyzing the material composition and the printing path provide two reasons for this phenomenon. Firstly, the layers’ cohesion is determined by the impregnated material or, alternatively, the fact that the fiber lining the XY plane cannot enhance mechanical strength in the stacking direction. Zhang et al. [15,16] observed that the nature of three-dimensional layer-by-layer printing results in the weakest bonding force between the layers. Therefore, in addition to increasing the strength of the CFRTPC filament itself, it is also necessary to study the printing path planning method by laying CFRTPCs to achieve better multiple-direction mechanical reinforcement. With regard to path algorithms, Wang et al. [17] proposed a five-axis slice algorithm to print the unsupported spiral pipe. Huang et al. [18,19] developed a curved layer slicing method that combined adaptive flat layer and curved layer slicing. Zhao et al. [20] studied an inclined layer printing strategy to print overhanging structures without a supporting structure. The main purpose of these algorithms is to accomplish unsupported printing whilst improving the surface quality of printed parts. There is little research on strengthening the bonding strength between layers. If CFRTPCs can be paved along the stacking direction, that is, the *Z*-axis direction of the printed parts, the problem of insufficient interlayer bonding can be resolved.

In order to achieve both layer-by-layer printing and printing along the stacking direction, traditional three-dimensional printing equipment is inadequate, so multi-axis printing equipment is essential. In recent years, various multi-axis printing equipment has been developed. Yvind Kallevik Grutle [21] designed a five-axis printer based on a 3D printing platform. Kyosuke Kawagishi et al. [22] developed a new four-axis 3D printer using FDM technology and selected the C-axis rotating around the *Z*-axis as the fourth axis. Steven Keating et al. [23] combined a robot arm with a traditional five-axis milling machine for use in the FDM process. Most studies have used a double nozzle (inc. Luan et al. [13], Markforged [24]) throughout three-dimensional machines. However, most multi-axis 3D printing equipment uses pure polymer materials, and CFRTPC printing has focused on conventional planar layer slicing printing. Combining CFRTPCs, double-nozzle, multi-axis and path algorithms may enhance the bonding performance between layers if CFRTPCs can be printed on the surface of formed polymer objects along the stacking direction.

In summary, research has been carried out on CFRTPC materials, the forming process, path planning algorithm and equipment development. The purpose of this paper is to study the printing path planning algorithm and develop double-nozzle five-axis printing equipment capable of printing CFRTPCs on the curved surface of printed parts and mechanically reinforcing along the stacking direction. In addition, this paper introduces pressure, tensile and bending tests to test the mechanical properties of the pipe samples and compare them with each other.

## 2. Experimental Platform and Materials

### 2.1. Experimental Platform

**Figure 1:**
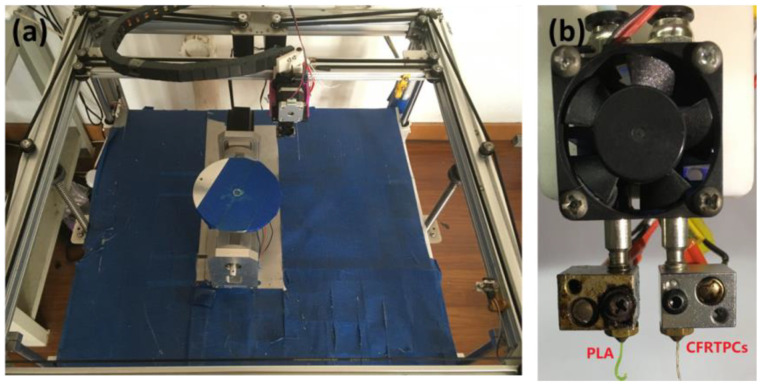
Experimental platform. (**a**) The five-axis platform. (**b**) The double nozzle.

A double-nozzle FDM printer for PLA and CFRTPC printing was designed and built in our laboratory [2]. The platform consisted of a receiving platform, X-Y-Z motion mechanism plus two rotation axes, and a double nozzle, as shown in Figure 1. To manufacture the pipe structure using CFRTPC printing, PLA was used to print the part base structure via the No.1 nozzle and then pave the fiber above the surface of the printed base structure. The No.1 nozzle is a conventional 0.4 mm nozzle, but the No.2 nozzle is a 1 mm nozzle whose fillet edge exerts downward force on the filament during printing. The bottom edge of the double nozzle is at the same horizontal height.

### 2.2. Materials

**Figure 2:**
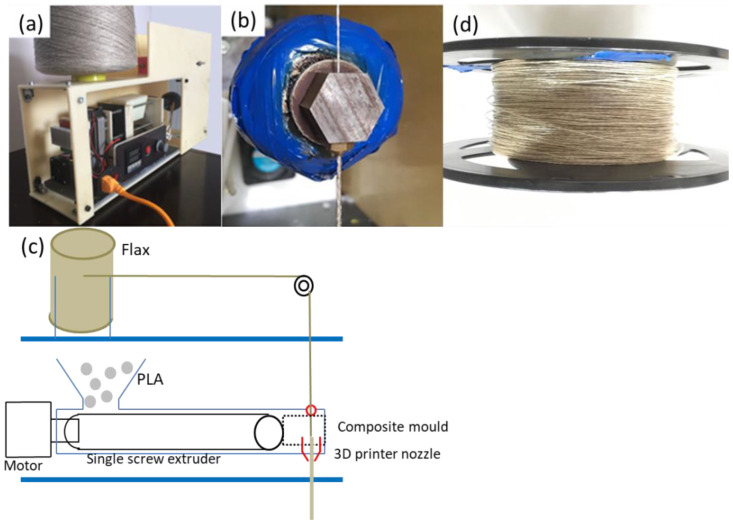
The production of the CFRTPCs. (**a**) The prepreg device. (**b**) The composite mold. (**c**) Schematic for manufacturing CFRTPC filaments. (**d**) The CFRTPCs after impregnation.

The PLA filament with a diameter of 1.75 mm was manufactured in JG Maker, and its density is 1.21 g/cm^3^ and the printable temperature range is 190~210 °C. The flax fiber provided by Kompetenzzentrum Holz GmbH in Austria was twined by two strands, and its diameter is 0.4 mm. CFRTPCs were prepared by impregnation using our laboratory equipment shown in Figure 2a. The PLA particles were heated to the molten state in the composite of Figure 2b, and the flax fiber entered through the hole above and was pulled out by the traction device below. The impregnated continuous flax fiber-reinforced PLA composites are shown in Figure 2d, and their diameter is 1 mm, so the fiber volume fraction of the composites is 16%.

### 2.3. The Double-Nozzle Setting

**Figure 3:**
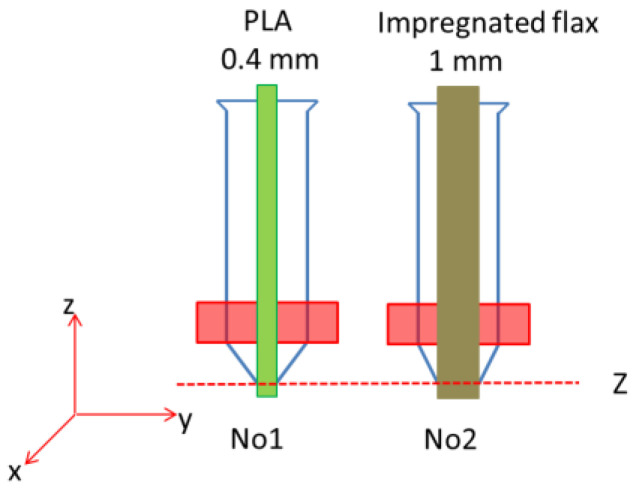
Double nozzle.

**Figure 4:**
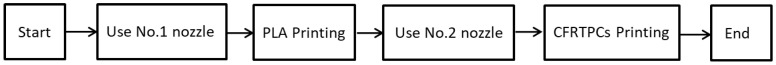
Schematic diagram of sequence of the use of materials.

As for the double-nozzle switch, the master control chip of the printer has an extensible extrusion module, so one extra extrusion module was added to drive another nozzle. T0 and T1 represent the No.1 nozzle and No.2 nozzle in Gcode, respectively. T0 drives the No.1 nozzle to print the base structure using PLA material, and T1 drives the No.2 nozzle to print the axial reinforcement section using CFRTPC material. Due to there being no height difference, x and y coordinates must add the displacement difference between the No.1 nozzle and No.2 nozzle during the T1 period, as shown in Figure 3. We set the extrusion temperature of the No.1 and No.2 nozzles to 195 °C, the PLA extrusion speed to 1000 mm/min and the CFRTPC deposition speed to 100 mm/min. The CFRTPC printing layer height is 0.5 mm, the filaments spacing is 1 mm and the number of printing layers is 1. In addition, Figure 4 shows the nozzles and filaments used during the printing process.

## 3. CFRTPC-Reinforced Printing Methodology

### 3.1. Part Modeling and Feature Recognition

**Figure 5:**
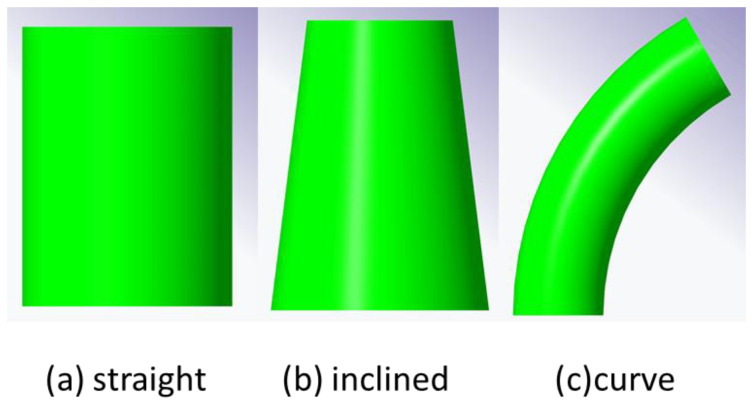
Three types of pipe.

This article uses pipe parts as an example. Bowls and bottles have a similar construction to pipes, namely curved surfaces and a hollow interior. In order to classify every type of pipe, the skeleton curve and cross-section area can be measured and analyzed. A skeleton curve can be used to distinguish a curved pipe from a straight pipe, and the cross-sectional area of every slicing layer can be used to distinguish between an inclined pipe and a straight pipe. Corresponding to the skeleton curve feature and pipe slicing layer cross sections, there are three common types, as shown in Figure 5. The former three pipes are generated by the 3D design software, and the pipe parameters such as diameter and height are designed according to the experimental requirements. The generated pipe STL data were used for printing path planning.

### 3.2. Technological Process 

**Figure 6:**
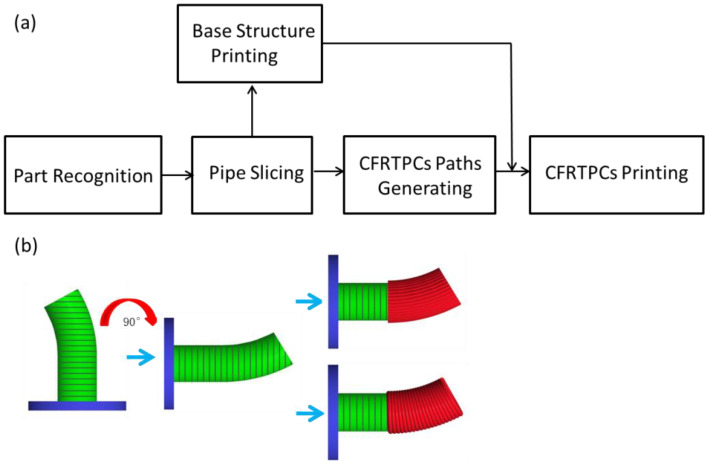
(**a**) Technological process of CFRTPC-reinforced printing methodology. (**b**) The hybrid printing flow.

The technological process of CFRTPC-reinforced printing methodology is shown in Figure 6a. Firstly, a pure polymer pipe is printed for use as a base structure; then, CFRTPCs are loaded onto the pipe surface, taking into account the printing paths. The skeleton curve perpendicular slicing is generated by the 5-axis dynamic slice algorithm [17], where every slicing plane is perpendicular to the skeleton curve. In addition, there are two CFRTPC paving styles due to differences in the cross-sectional area.

As shown in Figure 6b, firstly, a pure PLA pipe is printed as the base structure. Then, rotating the receiving platform enables the CFRTPCs to deposit on the base pipe surface. Lastly the CFRTPC printing is performed in two directions. The excess part under the base pipe is present to avoid interference between the extruder and the receiving platform.

### 3.3. Printing Path Planning

#### 3.3.1. Printing Path Planning of the Base Structure

**Figure 7:**
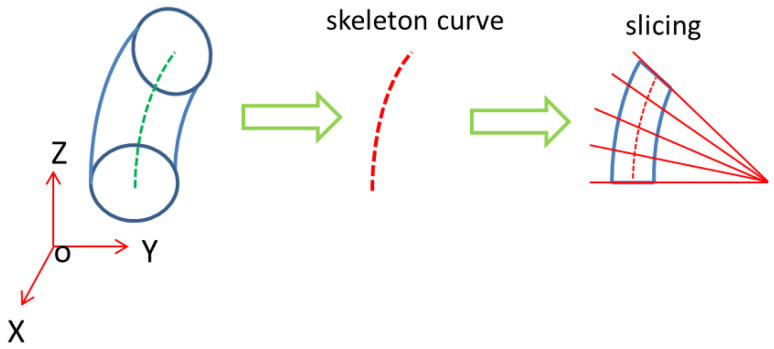
Base structure of curve pipe slicing.

**Figure 8:**
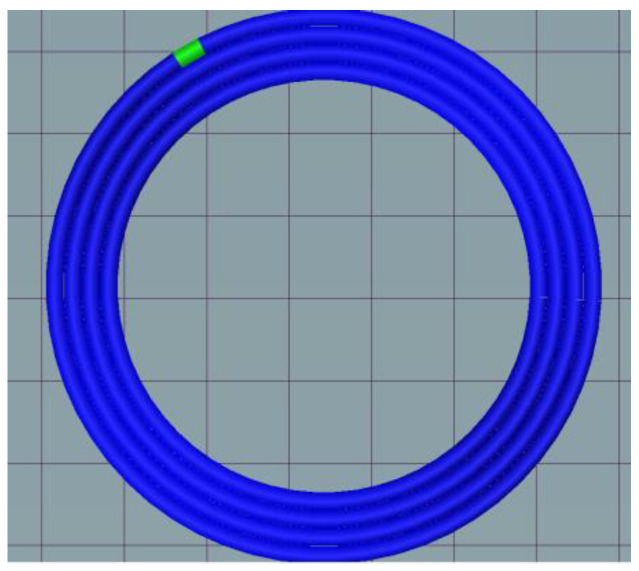
Concentric circle fill path of every slicing layer.

Slicing is essential for printing; when the skeleton curve of the pipe is a straight line, the traditional horizontal slicing method can be used without support. However, in a curved pipe, horizontal slicing will produce redundant supports which damage the surface quality of the part when removed. A 5-axis dynamic slice algorithm is appropriate when dealing with a curved pipe. The skeleton curve extraction of a curved pipe can be achieved by using the mean curvature flow algorithm [25]. The extraction process is shown in Figure 7. The skeleton curve is important later in the process as well as in slicing. Five-axis slicing copes with curved pipes, as every slicing layer is perpendicular to the tangent of the skeleton curve cross section. At the current slicing layer, concentric circle filled paths are created, as shown in Figure 8. Additionally, the points on the outermost circle are where later CFRTPCs are paved on. The printer can obtain the base structure printing path using this process.

#### 3.3.2. Printing Path Planning of CFRTPC-Reinforced Curve Surface

**Figure 9:**
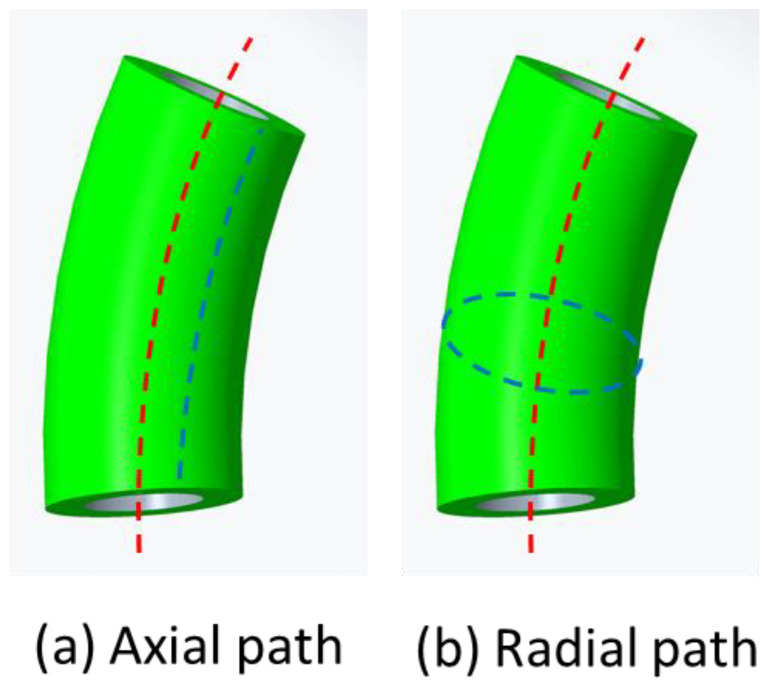
Two printing directions.

CFRTPCs are paved onto the pipe surface of the base structure. According to the printing direction, the printing path can be classified into two types: axial direction, where the path is parallel to the skeleton curve, and radial direction where the path is perpendicular to the skeleton curve; as an example, a curved pipe is shown in Figure 9.

**Figure 10:**
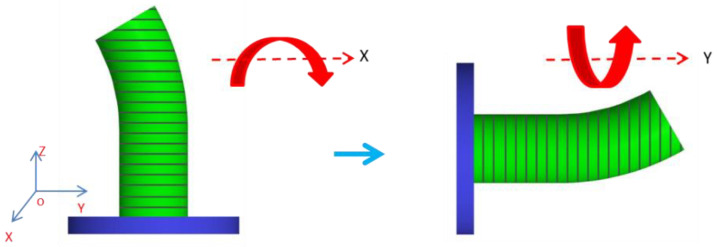
Rotation.

**Figure 11:**
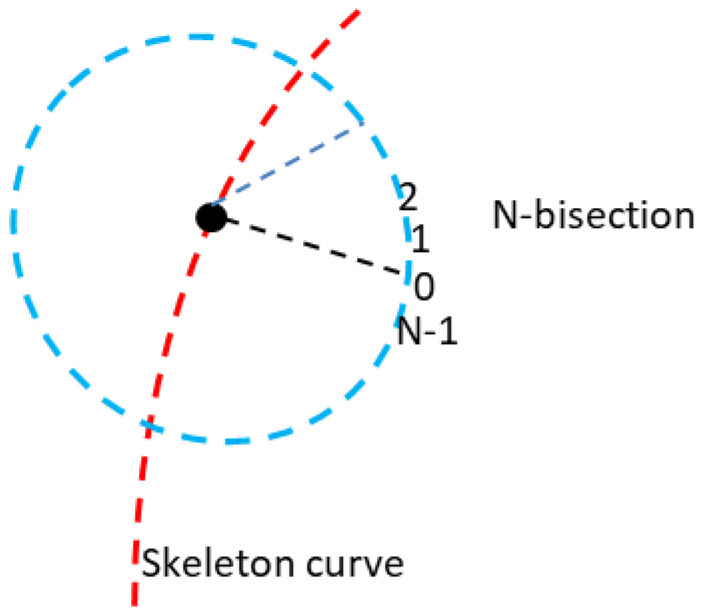
N-bisection.

Axial path: Firstly, the formed PLA pipe base placed vertically cannot be laid with CFRTPCs, so the receiving platform is set parallel to the *Z*-axis to let CFRTPCs be paved on the pipe surface by the translation matrix Tran_a_ (rotating the XY plane 90 degrees around the *X*-axis), as shown in Figure 10. Secondly, the outline of every layer is divided into N equal parts and arranged in order, as shown in Figure 11. Finally, the same serial number of every layer is connected to one axial path L_[i]_. Due to the receiving platform rotation around the *Y*-axis instead of the nozzle rotation to let the unprintable part of the pipe arrive at the printable area of the nozzle, every axial path L_[i]_ must rotate the corresponding angle (360 ∗ i)/N degrees around the *Y*-axis, namely the rotation matrix Rot_b_.

The translation matrix Tran_a_ to rotate to the Z plane is:

(1) $${\mathrm{Tran}}_{a}=\left[\begin{array}{cc} \begin{array}{cc} 1 & 0\\ 0 & cos90{}^{\circ} \end{array} & \begin{array}{cc} 0 & 0\\ -sin90{}^{\circ} & 0 \end{array}\\ \begin{array}{cc} 0 & sin90{}^{\circ}\\ 0 & 0 \end{array} & \begin{array}{cc} cos90{}^{\circ} & 0\\ 0 & 1 \end{array} \end{array}\right]$$

The rotation matrix Rot_b_ around the *Y*-axis is:

(2) $${\mathrm{Rot}}_{b}=\left[\begin{array}{cc} \begin{array}{cc} cos\frac{360\;*\;i}{N} & 0\\ 0 & 1 \end{array} & \begin{array}{cc} sin\frac{360\;*\;i}{N} & 0\\ 0 & 0 \end{array}\\ \begin{array}{cc} -sin\frac{360\;*\;i}{N} & 0\\ 0 & 0 \end{array} & \begin{array}{cc} cos\frac{360\;*\;i}{N} & 0\\ 0 & 1 \end{array} \end{array}\right]$$

Given one point P[i] on the outermost path, the final point coordinate P[i]_F_ is:

P[i]_F_ = p[i] • Tran_a_ • Rot_b_ (3)

Radial path: We also ensure that the receiving platform is parallel to the *Z*-axis. Figure 7 shows the skeleton curve point corresponding to the center of the outermost circle of every corresponding layer. Based on the skeleton curve expression and the outermost circle equation for every layer, we set the last point of the current layer as the starting point of the next layer. The displacement offset was then added to the other current layer point, and one spiral curve enveloping the surface of the current layer was fitted using the least-squares method. The line L_[f]_ connects every fitting curve of the layer and envelopes the pipe surface. Similarly, L_[f]_ is not the path the nozzle moves. Finally, we replaced the nozzle rotation with the receiving platform rotation. The matrix of both ways is the same, as all points are at the pipe surface, and only their connection order is different. 

**Figure 12:**
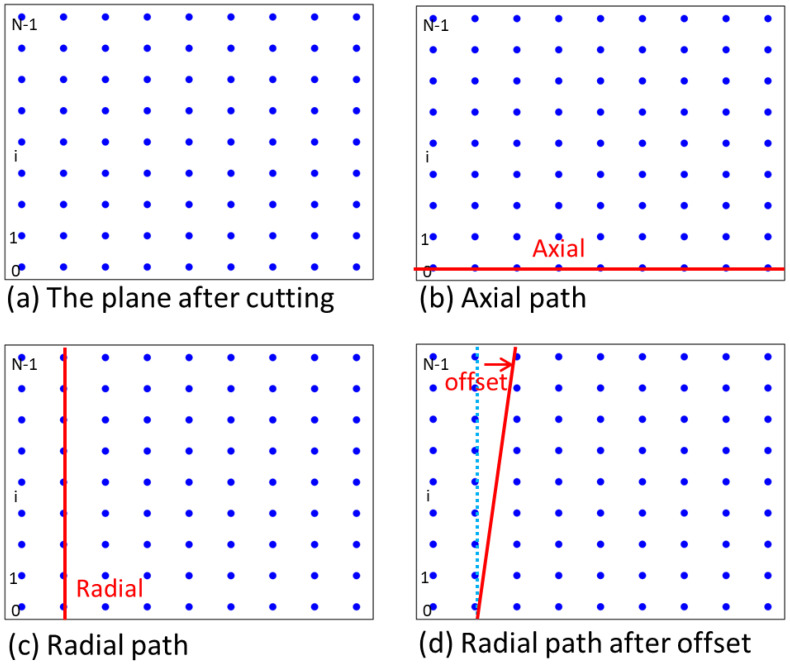
Path diagram.

A rectangular plane (namely the surface of the pipe) was obtained by cutting one straight pipe along its axial direction, and the blue points in Figure 12a display every point on the outermost circle. Figure 12b,c show the axial and radial paths by connecting every point. Additionally, adding the corresponding offset to every point of every circle will find a continual path, as shown in Figure 12d.

**Figure 13:**
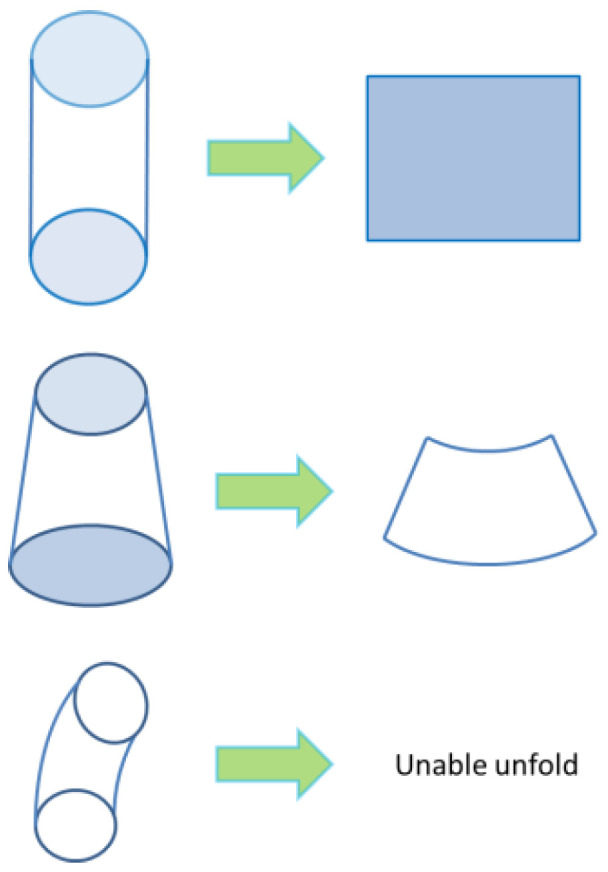
The unfolding drawing of pipes.

The unfolded inclined pipe drawing is one sector, and the axial path converges gradually as the radial path of every circle decreases. The radial path of the curved pipe is equal while its axial paths have a difference according to the bending radian, as shown in Figure 13. Although the curved pipe cannot be directly unfolded, every slicing layer can be unfolded as a straight pipe with unequal heights on both sides.

**Figure 14:**
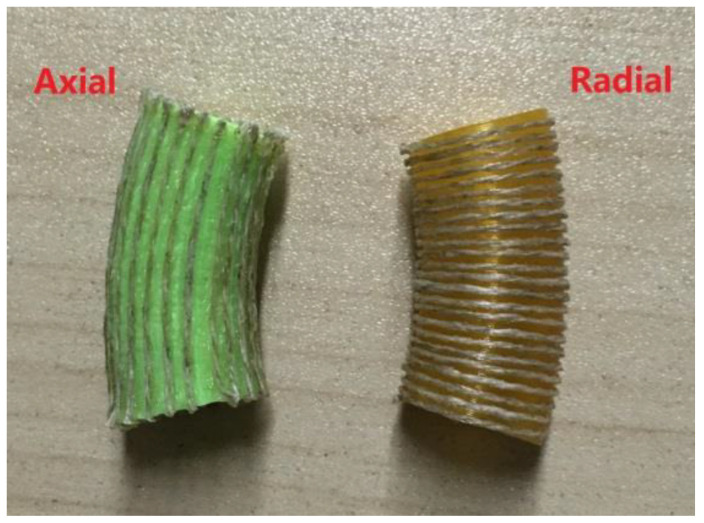
Axial and radial pipes.

Via the former process, we obtained axial and radial CFRTPC curved pipes, as shown in Figure 14.

## 4. Mechanical Property Test

All tests (pressure, tensile and bending) were performed using a WDW-1 microcomputer control electron universal testing machine. The test object was the straight pipe, and the printing parameters were the same as those in Section 2.

### 4.1. Pressure Test

**Figure 15:**
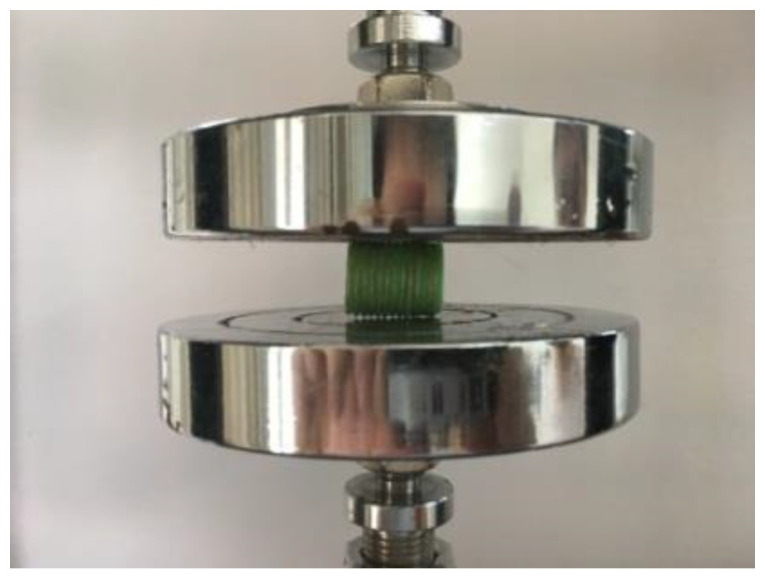
Compression test.

In order to avoid anisotropy and balance all forces, an ordinary straight pipe was used as a test subject. The height (h), inner diameter (r) and outer diameter (R) of the pipe are 20 mm, 14 mm and 15 mm, respectively, and its test specimen code was termed according to the r and R of the pipe. Hence, the codes were 1415, 1416 and 1417. In addition, the CFRTPCs were paved only axially and radially in 1415, and the other two were control groups. The destructive compression was carried out to test the CFRTPCs’ mechanical properties, as shown in Figure 15.

**Figure 16:**
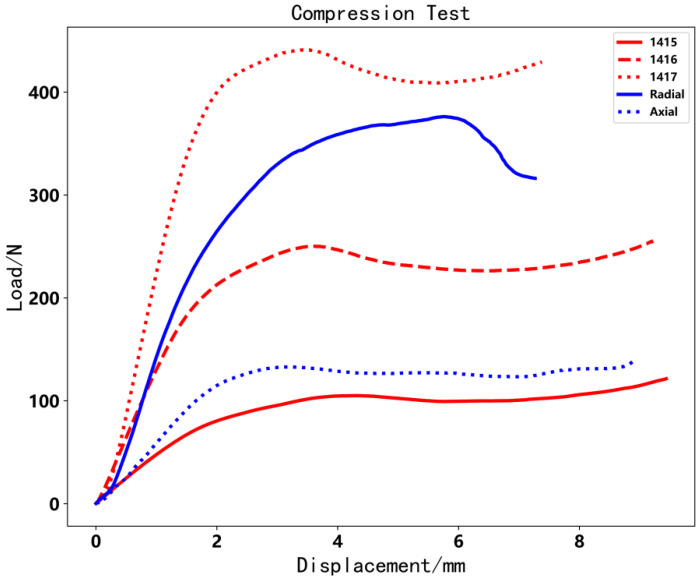
Load image of compression test.

Figure 16 shows that the load varies with displacement. With increasing displacement, the structure of the object is destroyed. Hence, we chose the first maximal testing load as the reference object. According to Table 1, the axial CFRTPCs do not have much effect on the compression direction. Nevertheless, radial CFRTPCs progress similarly to the 1417 specimen. The mass of the radial CFRTPC specimen is close to that of 1416. The radial and 1416 specimens both deposit 1 mm filament on the 1415 specimen. Compared to the control group, the max load of the radial specimen increased by 54% compared to the 1416 specimen.

### 4.2. Tensile Test

**Figure 17:**
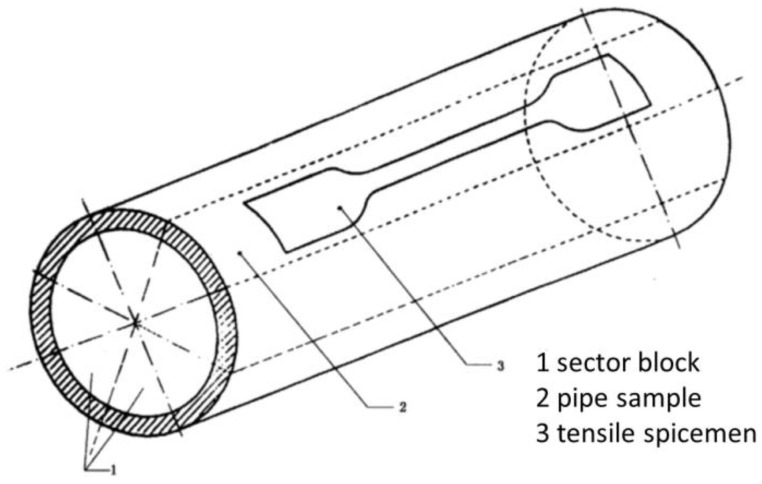
Tensile specimen fabrication.

**Figure 18:**
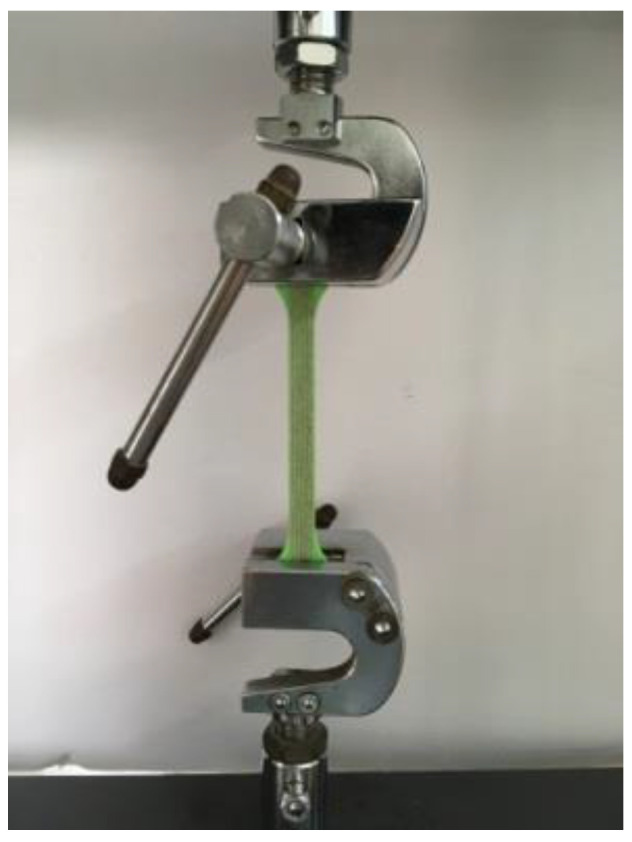
Tensile test.

According to ISO 6259-1:1997, to determine the tensile properties of thermoplastic pipes, sample pieces of a specified shape and size were made by longitudinal cutting or machining, as shown in Figure 17, and the total length is 150 mm, the gauge distance is 80 mm, the end width is 20 mm, the narrow width is 10 mm, and the radius at the arc is 60 mm. Then, a dog-shaped specimen was fabricated to provide uniaxial tension, as shown in Figure 18. In the meantime, 1415, 1416 and 1417 have thicknesses of 1 mm, 2 mm and 3 mm, and the CFRTPCs keep paving on the 1415 basis through both axial and radial directions.

**Figure 19:**
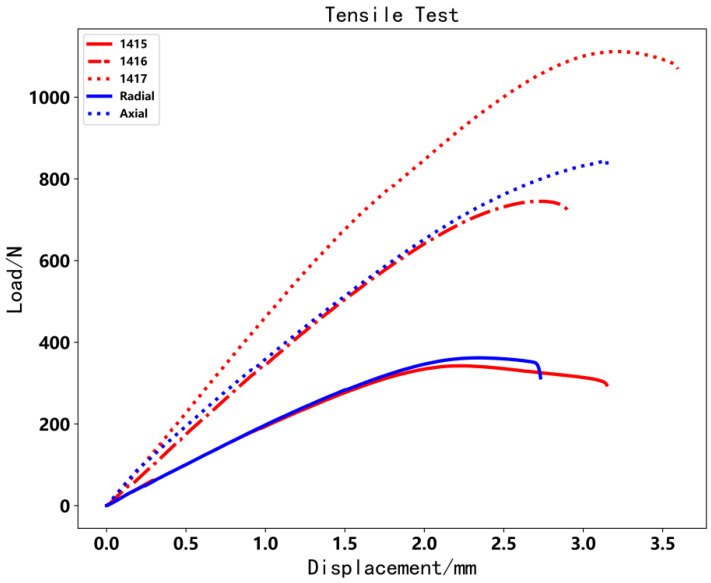
Load image of tensile test.

Figure 19 shows that the load varies with displacement. With increasing displacement, the specimen broke. We also chose the maximal testing load as the reference object. According to Table 2, radial CFRTPCs had no distinguishable effect on tensile direction, and the radial path revealed CFRTPCs deposition along *Z*-axis played a minor role in layer adhesion. However, axial CFRTPCs surpass specimen 1416 but were inferior to specimen 1417. Compared to the pressure test, the maximum load of the axial specimen increased by 13% compared with the 1416 specimen.

### 4.3. Three-Point Bending Test 

**Figure 20:**
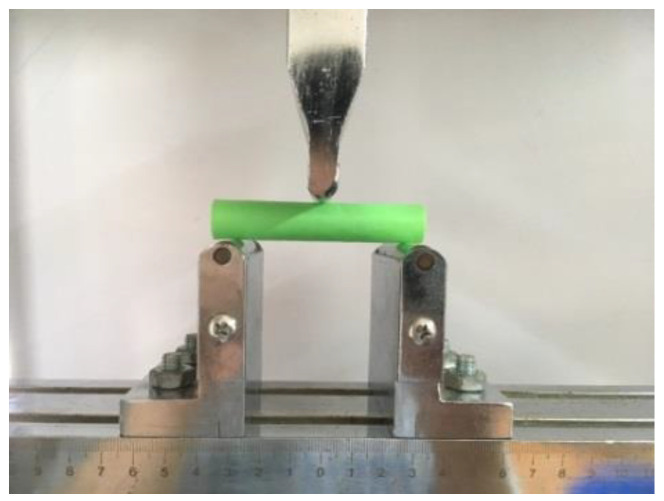
Three-point bending test.

Similar to the rectangular specimen (ISO 14125:1998, fiber-reinforced plastic composites, determination of flexural properties, NEQ) for a three-point bending test, we just increased the height of the straight pipe from 20 mm to 80 mm, as shown in Figure 20.

**Figure 21:**
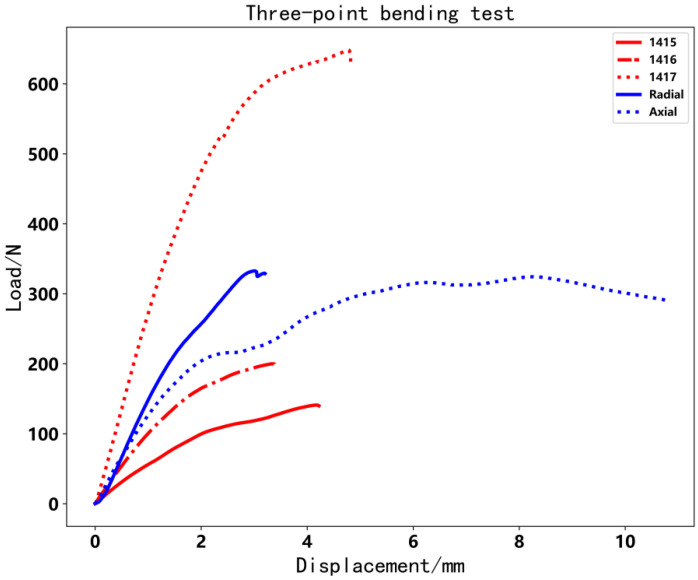
Load image of three-point bending test.

**Figure 22:**
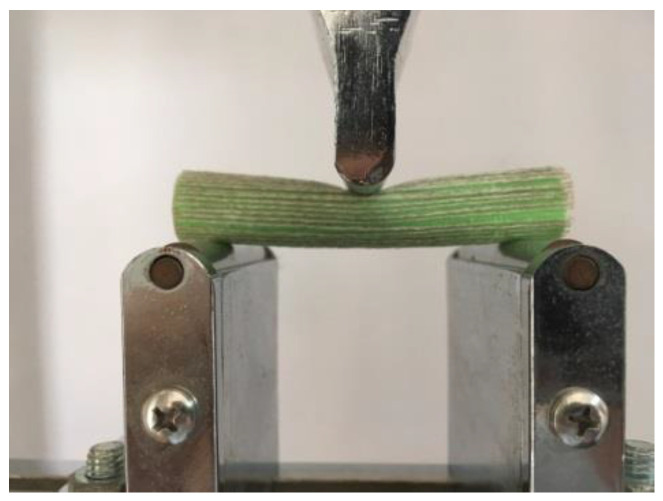
Deformation by load.

Figure 21 shows that the load varies with displacement and Table 3 shows the maximum load of every specimen. The load force of radial and axial CFRTPC specimens increased significantly. Additionally, the axial specimen generated a second phenomenon because the load object transfers from the point (or line) to the plane, as shown in Figure 22. The max load of the radial/axial type increased by about 70% compared with the 1416 specimen.

Through the above tests, there is no doubt about the properties of 13% flax fiber. Under the conditions of the same printing layer, that is, the thickness of the test pipe is the same, the radial path can enhance the compression and bending performance and the axial path tensile and bending performance. On the basis of the 1415 sample, compared with 0.5 mm-thick PLA, the compressive strength and bending strength of 0.5 mm CFRTPCs are 2.08 times and 3.17 times, respectively, and as for tensile properties, 0.5 mm CFRTPCs is 1.25 times higher than 1 mm-thick PLA. In addition, the printing method of mixing PLA and CFRTPCs greatly reduces the time required for pure CFRTPC printing, and the number of layers and path of CFRTPC printing can be set according to the performance requirements.

## 5. Conclusions

Given the weak mechanical performance of pipe sections along the *Z*-axis, this paper proposes a hybrid printing method of pure polymer material and CFRTPC material. Hybrid printing refers to the use of polymer (PLA) printing and fiber (CFRTPCs) printing, rather than the traditional printing method of compositing fiber and polymers. Firstly, a double-nozzle five-axis printer prints the base structure using PLA through slicing perpendicularly to the skeleton curve. Subsequently, the CFRTPC filament is paved along the axial and radial directions on the surface of the polymer base. Finally, the mechanical properties of the pipe specimens are investigated. The radial CFRTPCs can strengthen the compressive property of pipe parts whilst axial CFRTPCs enhance the tensile property. In addition, there is no difference in bending property between the radial and axial CFRTPCs from the final enhancement effect, but the rising process of enhancement effect is very different. Based on the 1415 pipe, 0.5 mm CFRTPCs can improve compression, tensile and bending properties by 250 N, 500 N and 190 N, respectively. The hybrid printing method compensates for the deficiencies of *Z*-axis adhesion whilst enhancing other performance aspects.

The slicing algorithm is suitable for axisymmetric pipe parts, but non-axisymmetric pipe parts need to be further researched. Pipes with curved surfaces are easier to print using an extruder that can rotate at particular angles. In addition, the master control chip used in this paper cannot meet a five-axis linkage. Future work should revolve around these issues.

## Figures and Tables

**Table 1:** Compression test results.

Test Code	1415	1416	1417	Radial	Axial
Maximal load/N	120	240	420	370	130
Mass/g	0.83	1.38	1.90	1.42	1.40

**Table 2:** Tensile test results.

Test Code	1415	1416	1417	Radial	Axial
Maximal Load/N	342.1	744.9	1111.4	361.7	844.5

**Table 3:** Three-point bending test results.

Test Code	1415	1416	1417	Radial	Axial
Maximal load/N	141.0	199.9	648.5	332.6	324.3
